# Comments on “Business intelligence for detecting possible surgical site infections from post-cesarean section operation with a focus on antibiotic prescriptions in Ramathibodi Hospital, Thailand”

**DOI:** 10.1017/ash.2025.10246

**Published:** 2025-12-12

**Authors:** Sushma Narsing Katkuri, Varshini Vadhithala, Arun Kumar, Sushma Verma, Dhanya Dedeepya

**Affiliations:** 1 Department of Community Medicine, Malla Reddy Institute of Medical Sciences, Malla Reddy Vishwavidyapeeth, Hyderabad, Telangana, India; 2 Dr. D. Y. Patil Medical College, Hospital and Research Centre, Dr. D. Y. Patil Vidyapeeth (Deemed-to-be-University), Pune, Maharashtra, India; 3 Faculty of Pharmaceutical Sciences, Graphic Era Hill University, Dehradun, India; 4 Centre for Promotion of Research, Graphic Era Deemed University, Dehradun, India; 5 Department of Pharmaceutics, Noida Institute of Engineering & Technology (Pharmacy Institute), Greater Noida, India; 6 Saveetha Medical College and Hospital, Saveetha Institute of Medical and Technical Sciences, Saveetha University, Chennai, India; 7 Center for Innovation and Inclusive Research, Sharda University, Greater Noida, India

Dear Editor,

The authors should be commended for applying routinely collected hospital data to streamline surveillance for postcesarean surgical site infection (SSI). Their focus on operational workload and diagnostic performance is timely and aligns with current efforts to embed surveillance into electronic systems. Nevertheless, several methodological aspects warrant clarification to ensure appropriate interpretation of their findings.

First, the study states that SSIs were defined according to the NHSN 30-day postsurgical window, yet patients were followed for 45 days, and the “optimal” antibiotic-based surveillance rule was identified as days 8–45 after surgery. This creates ambiguity about the true reference period: whether the gold-standard adjudication allowed SSI onsets beyond day 30 or whether the extended follow-up was only to detect late diagnoses of events that actually began within 30 days. Diagnostic accuracy estimates are highly sensitive to the alignment between the target condition and the time window used to define both index test and reference standard. An explicit statement of onset criteria, and ideally a sensitivity analysis restricted to the strict 30-day period, would strengthen internal validity.

Second, the infection control nurse review is treated as an independent gold standard, yet it is informed by the same antibiotics, microbiology, and coding data that underpin the business-intelligence (BI) algorithm. This “incorporation bias” can inflate sensitivity and specificity by making the reference standard partly dependent on the index test. Contemporary guidance on diagnostic-accuracy studies recommends either an independent reference or, when that is not feasible, explicit acknowledgment and discussion of this limitation. Future work might consider additional clinical validation (eg, chart review blind to BI flags) in a subset of cases.

Third, the workload comparison is described as a paired-sample design analyzed with the Wilcoxon signed-rank test, yet only aggregate “before–after” counts and times over three months are reported. Without clearly defined pairing units (such as weekly or daily workloads), the stated test may not correspond to the actual data structure. Transparent reporting of the unit of analysis and the number of paired observations is essential to interpret p-values and effect sizes appropriately.

These issues do not negate the promising potential of BI-assisted SSI surveillance demonstrated by the authors, but they do suggest that the reported performance metrics may represent optimistic upper bounds. Clearer specification of the surveillance window, explicit handling of incorporation bias, and more detailed reporting of the workload analysis would enhance confidence in the generalizability and reproducibility of this approach.

